# Commentary: Immunogenic Cell Death and Immunotherapy of Multiple Myeloma

**DOI:** 10.3389/fcell.2019.00149

**Published:** 2019-07-31

**Authors:** Ken Maes, Karine Breckpot

**Affiliations:** ^1^Department of Hematology and Immunology-Myeloma Center Brussels, Vrije Universiteit Brussel, Brussels, Belgium; ^2^Laboratory for Molecular and Cellular Therapy, Vrije Universiteit Brussel, Brussels, Belgium

**Keywords:** multiple myeloma, immunogenic cell death, immunotherapy, immunogenicity, tolerance

In April 2019, Serrano-del Valle and colleagues summarized the current applications of immunotherapy in the plasma cell malignancy multiple myeloma (MM) (Serrano-Del Valle et al., 2019). The authors moreover highlighted the connection of endoplasmic reticulum (ER) stress and the occurrence of immunogenic cell death (ICD). ICD is able to provoke potent adaptive immune responses and is based on the release of danger-associated molecular patterns (DAMPs) in a spatiotemporal manner (Garg et al., 2015). Interestingly, ER-stress pathways are important for DAMP exposure, including ecto-calreticulin, HMGB1, and ATP (Serrano-Del Valle et al., 2019). MM cells display enhanced ER-stress, hence making them dependent on ER-stress-related survival pathways. Compounds that target these survival pathways or that induce excessive amounts of ER-stress are very effective in targeting MM cells. Therefore, it is tempting to speculate that ICD is an important mode of action of standard-of-care treatment.

To assess the occurrence of ICD *in vivo*, a vaccination assay can be performed in which dying tumor cells are used as a vaccine in mice (Kepp et al., 2014). In case *bona fide* ICD occurs, mice will be protected against a challenge with living tumor cells. We were the first to perform this vaccination assay using the syngeneic immunocompetent 5T33MM model (De Beck et al., 2018). The vaccine consisted of 5T33vt cells treated with bortezomib, melphalan, a DNA methyltransferase inhibitor (decitabine), a histone deacetylase inhibitor (quisinostat) or the combination of decitabine and quisinostat. We also used a well-known chemotherapeutic compound that induces ICD in solid cancers, i.e., mitoxantrone (Emeagi et al., 2012; Bezu et al., 2015). None of these treatments gave rise to a vaccine that provided 100% protection against MM outgrowth upon a subsequent challenge with living MM cells. This indicates that ICD did not occur in this model. When looking at the concept of ICD, it is important to note that *bona fide* ICD can only occur when tumor cells can expose the necessary DAMPs in response to treatment and when the recruited immune cells are not compromised in their function. However, when tumor cells cannot present all DAMPs, express tolerogenic molecules, or when immune cells cannot react properly on the DAMPs, ICD becomes inefficient resulting in tolerogenic cell death (TCD) (Garg et al., 2016). The occurrence of ICD in MM is influenced by the balance of tolerogenic and immunogenic molecules and the associated receptors which are expressed by tumor and immune cells in the bone marrow (BM). In our study, we addressed DAMP exposure in 5T33vt cells. We showed that ecto-calreticulin was present on a low amount of treated pre-apoptotic cells and that the “don't eat me signal” CD47 was highly expressed at basal and treatment conditions. None of the compounds increased the release of HMGB1. Decitabine, quisinostat, and melphalan induced a type I interferon response and induced signs of dendritic cell (DC) maturation upon co-culture of treated 5T33vt cells with BM monocyte-derived DCs. *In vivo*, epigenetic-modulating compounds increased ecto-calreticulin and decreased CD47 expression in tumor cells, increased DC maturation, reduced CD11b-positive myeloid cells and transiently increased the amount of memory and naive T cells in the BM. Unfortunately, we could not unambiguously show that ICD occurred in the 5T33MM model as the vaccination assay did not provide 100% protection.

Other studies also focused on potential ICD effects as a mode-of-action of standard-of-care compounds for MM patients and other compounds in pre-clinical studies (Table 1). Bortezomib induced an adaptive immune response *in vitro* with U266 and patient-derived MM cells in a HSP90 dependent manner (Spisek et al., 2007; Moeller et al., 2012). Carfilzomib treatment exposed calreticulin in 7-AAD-negative human myeloma cells (Jarauta et al., 2016). Alkylating agents, including melphalan and cyclophosphamide, induced hallmarks of ICD including ecto-calreticulin and HMGB1 in thymoma, lymphoma, and colorectal cancer models (Schiavoni et al., 2011; Dudek-Perić et al., 2015; Lu et al., 2015). Other immune-related effects were also described, including depletion of regulatory T cells, induction of type I interferon, and increased effectiveness in conjunction with adoptive T cell transfer (Condomines et al., 2010; Sharabi and Haran-Ghera, 2011; Moschella et al., 2013). In MM, melphalan induced exosome release and Natural-Killer cell cytokine production in an HSP70-dependent manner (Vulpis et al., 2017). The fatty acid docosahexaenoic acid (DHA) increased ecto-calreticulin and HMGB1 release (D'Eliseo et al., 2017). Moreover, DHA-treated cells stimulated signs of maturation of *ex vivo*-generated DCs. The combination of a miR34 mimic and a gamma-secretase inhibitor induced exposure of calreticulin in MM cell lines (Zarone et al., 2017). At last, the IAP antagonist LC161 increased phagocytosis and induced a type I interferon response and long-lasting anti-MM immunity, independently of the presence of ecto-calreticulin (Chesi et al., 2016).

**Table 1:** Current evidence of ICD hallmarks exposed by multiple myeloma cells.

**References**	**Compound**	**Compound class**	**ICD-hallmark**	**Stage**	**MM model**	**Species**
Spisek et al., 2007; Moeller et al., 2012	Bortezomib	Proteasome inhibitor	HSP90	Early/Mid-apoptotic	Cell line (U266)	Human
					Primary MM cells	Human
Chesi et al., 2016	LC161	IAP antagonist	Type I IFN	N.D.	Cell line (Vk14451)	Mouse
					Primary MM cells	Human
Jarauta et al., 2016	Bortezomib and carfilzomib	Proteasome inhibitor	Ecto-Calreticulin	Early/Mid-apoptotic	Cell lines (U266, NCI-H929, and MM.1S)	Human
Vulpis et al., 2017	Melphalan	Alkylating agent	HSP70 on exosomes	N.D.	Cell lines (SKO-007 and ARK)	Human
D'Eliseo et al., 2017	Docosahexaenoic acid	Fatty Acid	HSP90	Early/Mid-apoptotic	Cell lines (OPM2 and RPMI8226)	Human
			Ecto-Calreticulin		Cell lines (OPM2 and RPMI8226)	Human
			HMGB1	Late-apoptotic	Cell lines (OPM2 and RPMI8226)	Human
Zarone et al., 2017	miR34a mimic and gamma-secretase inhibitor	miRNA mimetic and NOTCH modulator	Ecto-Calreticulin	N.D.	Cell line (RPMI-8226)	Human
De Beck et al., 2018	Melphalan	Alkylating agent	Type I IFN	N.D.	Cell line (5T33vt)	Mouse
			Ecto-Calreticulin	Pre-apoptotic	Cell line (5T33vt)	Mouse
	Decitabine, Quisinostat	Epigenetic-modulating compound	Type I IFN	N.D.	Cell line (5T33vt)	Mouse
			Ecto-Calreticulin	Pre-apoptotic	Cell line (5T33vt)	Mouse
	Bortezomib	Proteasome inhibitor	Ecto-Calreticulin	Pre-apoptotic	Cell line (5T33vt)	Mouse
	Mitoxanthrone	Type II topoisomerase inhibitor	Ecto-Calreticulin	Pre-apoptotic	Cell line (5T33vt)	Mouse

*N.D, not determined*.

Despite these studies, progress on identifying hallmarks of ICD is limited and the ICD-inducing capacities of MM cells needs to be better defined. The question thus arises whether standard-of-care agents in MM evoke strong anti-MM responses, especially because in MM, there is an inverse correlation between clinical outcome and mutational load, a parameter that at least in solid cancers is indicative for the success of many immunotherapies (Miller et al., 2017; Vitale et al., 2019). Furthermore, monocytes from MM patients show reduced efferocytosis (Liang et al., 2018). Monocyte-derived DCs from MM patients are ineffective in priming potent immune responses and plasmacytoid DCs have a pro-tumor phenotype by dampening T-cell responses rather than stimulating them (Bi et al., 2018; Shinde et al., 2018). Therefore, detailed examination of all molecular hallmarks of ICD and tolerogenic molecules on MM and immune cells in response to (combinations of) proteasome inhibitors, alkylating agents, immunomodulatory agents, dexamethasone, and monoclonal antibodies is warranted. This should be correlated to underlying stress responses (ER-stress and autophagy) or genetic alterations in a larger number of human MM cell lines, immunocompetent murine models and MM patient tumor and immune cells to obtain the broader picture of ICD and TCD. This is important as a misbalance between ICD and TCD toward TCD blunts therapy-induced immune priming and antitumor immune responses resulting in a worse outcome for patients.

## Author Contributions

All authors listed have made a substantial, direct and intellectual contribution to the work, and approved it for publication.

### Conflict of Interest Statement

The authors declare that the research was conducted in the absence of any commercial or financial relationships that could be construed as a potential conflict of interest.
